# Targeted Therapies in Adult B-Cell Malignancies

**DOI:** 10.1155/2015/217593

**Published:** 2015-09-06

**Authors:** Jean-François Rossi

**Affiliations:** ^1^Department of Hematology, University Hospital, CHU Saint Eloi, 80 avenue Augustin Fliche, 34295 Montpellier Cedex 05, France; ^2^Université Montpellier I, UFR Médecine, 34396 Montpellier, France

## Abstract

B-lymphocytes are programmed for the production of immunoglobulin (Ig) after antigen presentation, in the context of T-lymphocyte control within lymphoid organs. During this differentiation/activation process, B-lymphocytes exhibit different restricted or common surface markers, activation of cellular pathways that regulate cell cycle, metabolism, proteasome activity, and protein synthesis. All molecules involved in these different cellular mechanisms are potent therapeutic targets. Nowadays, due to the progress of the biology, more and more targeted drugs are identified, a situation that is correlated with an extended field of the targeted therapy. The full knowledge of the cellular machinery and cell-cell communication allows making the best choice to treat patients, in the context of personalized medicine. Also, focus should not be restricted to the immediate effects observed as clinical endpoints, that is, response rate, survival markers with conventional statistical methods, but it should consider the prediction of different clinical consequences due to other collateral drug targets, based on new methodologies. This means that new reflection and new bioclinical follow-up have to be monitored, particularly with the new drugs used with success in B-cell malignancies. This review discussed the principal aspects of such evident bioclinical progress.

## 1. Introduction

B-lymphocytes are programmed for immunoglobulin (Ig) production directed against pathogens via the B-cell receptor (BCR) activation. During this maturation process, B-lymphocytes exhibit different surface markers, activation of intracellular pathways, metabolism modulation, protein synthesis, and interaction with their microenvironment. B-lymphocyte ontogeny takes place in lymphoid organs leading to plasma cells that migrate into the bone marrow or mucosa-associated tissues. Recently, progress in biology knowledge has resulted in a large number of targeted therapies, designed against surface markers, cell signaling pathways, cell cycle and apoptosis machinery, key molecules involved in cellular metabolism, in proteasome, and in immune modulation, and niche disruption. Rituximab, an anti-CD20 monoclonal antibody (mAb), was the first targeted therapy successfully developed in lymphoma and chronic lymphocytic leukemia of B-cell type (B-CLL) [1–3]. A synergy with chemotherapy was demonstrated in all B-cell malignancies (B-CM) expressing CD20 molecules, with significant prolongation of the progression-free survival (PFS) and overall survival (OS) [4]. Beyond rituximab, there are new molecules directed against several factors. This includes (1) other surface markers, including not only other B-cell markers but also receptors (R) of survival/growth factors, such as B-cell activating factor/A proliferation-inducing ligand (BAFF/APRIL)R, interleukin (IL) 6R, IL7R, vascular endothelial growth factor (VEGF)R, epithelial growth factor (EGF)R, stromal cell-derived factor- (SDF-)1R or chemokine receptor type 4 (CXCR4), and insulin-like growth factor (IGF)1R; (2) key points for signaling pathways such as inhibitors of Bruton's tyrosine kinase (BTK), phosphoinositide 3-kinase (PI3K), and spleen tyrosine kinase (Syk); (3) inhibitors of cell cycle regulators; (4) proteasome inhibitors and nuclear factor kappa-B (NF*κ*-B) inhibitors; (5) metabolism inhibitors, such as antilacticodehydrogenase (LDH); (6) immune modulators; and (7) inhibitors of the interaction between tumor cells and its microenvironment. The complexity of these therapeutic options requires new reflection and approach and new drug combinations based on biological data in order to create optimal conditions for such new age in personalized medicine, including methodologies, follow-up to evaluate quality of life, and safety and tolerability not only just after the administration, but also after a long treatment period due to improved survival [5] (Tables 1, 2, and 3).

## 2. Cell Surface Markers

### 2.1. B-Cell Markers

**Figure 1:**
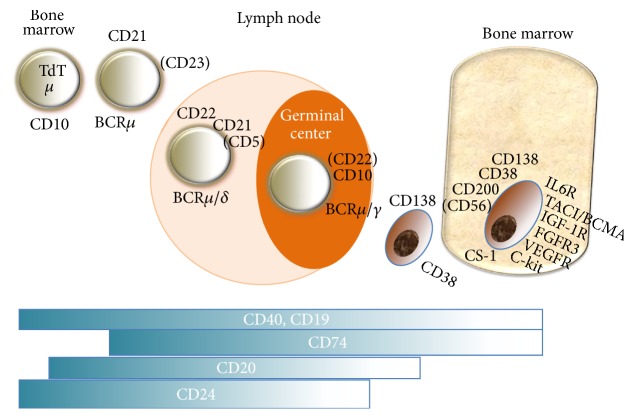
Surface markers of B-cell lineage present at the principal stages of differentiation, as targets for therapy. TdT: terminal deoxynucleotide transferase. TACI/BCMA: transmembrane activator and CAML interactor/B-cell maturation antigen. IGF-1R: insulin growth factor-1 receptor. EGFR: epithelial growth factor receptor. VEGFR: vascular endothelial growth factor receptor. IL6R: interleukin 6 receptor. FGFR3: fibroblast growth factor receptor type 3. c-Kit: CD117. BCR: B-cell receptor.

B-lymphocyte differentiation is associated with the expression of a variety of cell surface markers, including CD19, CD20, C22, CD40, surface Ig, and BAFF-R present at the different maturation steps, excluding the end-stage of this B-cell lineage, plasma cells [6–9]. Conversely, plasma cells also share CD19 and other surface markers such as CD38, CD138, CS-1, CD200, CD56, CD45, and other different markers [10] (Figure 1). In addition, all markers are currently used to define normal and malignant plasma cells, thus allowing evaluation of minimal residual disease, and to establish the true complete response (CR) expressed by the return of normal plasma cells inside the bone marrow niche [11]. Targeting surface B-cell markers also leads to cell signaling, as observed with CD19, CD20, CD5, and CD22 that are B-cell receptor (BCR) coreceptors with either stimulatory or inhibitory activities.

Rituximab, the first anti-CD20 mAb used in humans, has been shown clinically beneficial in B-CM, including non-Hodgkin's lymphoma (NHL) and B-CLL [1–3, 12]. This agent was also developed as a maintenance therapy with a significant prolongation of the therapeutic response. However, empirism was associated with the early development of rituximab, and the usual dose of 375 mg/m^2^ was not chosen on a biological basis. The choice of the efficient dose based on biological criteria was only made in the context of B-CLL, with the demonstration of biological efficacy through the quantification of the CD20 molecules at the cell surface and the engagement of the Antibody-Dependent Cell Cytotoxicity (ADCC) [13]. Maintenance therapy with rituximab was rather based on commercial reasons even though treatment prolongation for two years was associated with an improved PFS. In fact, treatment prolongation should have been based on the control of residual disease associated with improved survival. The dose was similar to the one used in chemoimmunotherapy, but with different options for the administration schedule due to a lack of biological rationale, except that ADCC with Natural Killer (NK) cell activation may delay the time of the optimal response.

Delayed complications have been neglected in this context. The first observations were made in patients treated for Rheumatoid Arthritis (RA) who experienced reduction of immune response to vaccination [14] and reactivation of viral infections, not only hepatitis also observed in the context of B-CM, but also progressive multifocal leukoencephalopathy (PML), a lethal encephalitis caused by the polyomavirus JC [15]. We recently observed one case of PML with a severe immune defect due to chemoimmunotherapy and autologous transplantation for NHL, 15 years after the initiation of the therapy (personal data). The fact that similar observations were not so frequent was due to three factors: (1) lack of substantial long-term survivors; (2) the patients having RA had a more pronounced immunodepressed status due to the exposition to several immune modulators, such as corticotherapy, methotrexate, or anti-Tumor Necrosis Factor (TNF) for example; and (3) relative limited efficacy of rituximab in depleting memory B-cells and plasma cell compartment within lymphoid organs [16]. This is not probably the end of the story and longer observation period is needed, particularly with improved efficacy and prolonged patient survival due to new efficient molecules, including the more efficient anti-CD20 mAbs and new targeted therapies. The subcutaneous (SC) form of rituximab was developed as equivalent to the IV formulation. However, the lymph node compartment being the target organ after SC administration was not considered. Had this been taken into account, one could predict a better activity and a better clinical use of the drug. The therapeutic strategy should change, and the current long-term maintenance therapy with rituximab should be avoided. In addition, drug agencies have to prolong patient observation beyond therapeutic response and to analyze the immune response with functional markers, for example, after vaccination [17]. Considerable progress was made in understanding the structure and the functions of CD20 molecules and anti-CD20 mAbs. Binding of the mAbs to their target supports three types of action: intracellular signals leading to programmed cell death, binding to C1q molecules inducing complement-mediated cell lysis, and Fc/FcR interaction or antibody-dependent cell cytotoxicity, particularly with NK lymphocytes [7]. Rituximab, Yttrium-90 ibritumomab, iodine-131 tositumomab, and ofatumumab are all anti-CD20 mAbs approved for different indications and countries, while others are used in clinical trials [7]. Yttrium-90 ibritumomab is an effective therapeutic agent for lymphoma, particularly in the treatment consolidation after immunochemotherapy induction as a first-line treatment for large B-cell lymphoma [18].

As CD19 is expressed by the B-cell lineage, from pro/pre-B-cells to plasma cells, anti-CD19 mAbs may represent good candidates for the treatment of B-CM [8]. Blinatumomab is a bispecific T-cell engager that specifically targets CD19 and CD3 antigens. This bispecific mAb was approved in December 2014 for Acute Lymphoblastic Leukemia (ALL) in USA [19]. In addition, CD19 was used as engineered receptors grafted onto immune effector cells, particularly on T-cells, to generate chimeric antigen receptor T-cells (CAR-T) that express a fusion protein comprised of an anti-CD19 mAb with CD28 costimulatory and CD3-*ζ* chain signaling domain. This novel technology was developed as adoptive transfer of CAR-T for ALL of B-cell type [20].

The success of rituximab has encouraged developers to propose other mAbs targeting different surface B-cell markers, such as anti-CD22 inotuzumab ozogamicin (CMC-544) or epratuzumab, combined with rituximab [21–23], anti-CD37 particularly for B-CLL [9], and anti-CD74 directed against a component of the HLA DR (milatuzumab) [12, 24]. Epratuzumab induces a marked decrease of CD22, CD19, CD21, and CD79b molecules on the B-cell surface and immune modulation on Fc*γ*R-expressing monocytes, NK cells, and granulocytes via trogocytosis [25]. Downstream the receptor, immune signaling involves specific tyrosine residues that are phosphorylated upon receptor activation. These phosphorylation sites are frequently found in one of the three types of Immunoreceptor tyrosine-based regulatory motifs (ITRM), including IT activation M (ITAM), IT inhibition M (ITIM), and IT switch M (ITSM) for SLAM/CD150 and related receptors of the CD2 subfamily [26]. Generally, ITIMs recruit the SH2 domain-containing tyrosine phosphatase SHP-1 or SHP-2, and phosphorylated ITAMs are recognized by SYK in B-cells [27, 28].

Epratuzumab combined with rituximab was associated with a high response rate including 42.4% of CR rate with 60% of the patients having 3-year remission, for untreated patients with follicular lymphoma (FL) [21]. This relatively high response rate is not superior to that observed with other treatments, but it opens the pathway for targeted therapy without chemotherapy. However, the combination of two mAbs is less cost-effective compared to new targeted drugs used orally; a decision was made to discontinue its development. A possible way for such development would be radioimmunotherapy and utilizing Yttrium-90 epratuzumab or other combination of CD22 with calicheamicin, or with PE38, a fragment of* Pseudomonas* exotoxin or novel anti-CD22 mAb that blocks CD22 ligand binding, or second generation ADCC with linkers and more potent toxins, particularly tried in ALL [22, 23].

CD19, CD200, CD38, CD138, CD56, and CS-1 are major targets expressed on Multiple Myeloma (MM) cells. MAbs against such molecules have been clinically developed [29]. Elotuzumab, a humanized mAb IgG1 antibody that targets CS-1 (SLAMF7), a cell surface glycoprotein with major expression in MM cells, has been shown to support very active ADCC [30]. It has been combined with lenalidomide and dexamethasone in patients having relapsed MM with promising results, 90% of the patients achieving a partial response (PR) with PFS exceeding 2 years [30]. A Phase III clinical study is ongoing and is due to be completed by 2017. Daratumumab is a humanized antibody against CD38 [31], a cell surface protein strongly expressed in MM [32]. CD38 is also expressed on malignant cells from B-CLL, mantle cell lymphoma (MCL), transformed FL, and clinical trials are ongoing with daratumumab in these diseases [31]. Some MM cells expressed CD56 and lorvotuzumab, an mAb against CD56, conjugated to mertansine has been developed in early clinical studies for MM [33]. CD200 is an immunosuppressive molecule overexpressed in several hematological malignancies including B-CLL, MM, and acute myeloid leukemia (AML) [34, 35]. Early clinical trials are ongoing in these diseases or in different models of immunotherapeutic strategies in AML [35]. Syndecan-1 (CD138) belongs to heparan sulfate proteoglycans that is highly expressed at the cell surface of MM cells [32, 36]. In addition, cell surface CD138 acts as a functional coreceptor for chemokines and growth factors in the plasma cell niche. Soluble form of syndecan-1 can accumulate survival factors within the microenvironment, representing a sort of sponge for these factors around the tumor cells [36]. Therefore, targeting this molecule is of potential clinical interest, due to a mixed activity on both the tumor cells and its cell niche, making the molecule attractive for radioimmunotherapy [37]. Different mAbs have been developed in early clinical phases including anti-CD40 mAbs such as lucatumumab, dacetuzumab, or mAb directed against HM1.24, the XmAb 5592 [38]. A total of 91 studies with mAbs are registered (https://clinicaltrials.gov/ct2/results?term=monoclonal+antibodies+in+multiple+myeloma&Search=Search) in MM patients, as of March 13, 2015.

For all of these mAbs and similarly to rituximab, clinical efficacy was only observed with combination strategies, particularly with other active drugs, depending on the response or the refractoriness to prior therapies, including bortezomib, IMiDs such as lenalidomide plus dexamethasone, and other new active drugs including approved molecules such as pomalidomide and carfilzomib, or other new targeted molecules.

### 2.2. Survival/Proliferation Factor Receptors

Upon recognition of foreign antigens, mature naive B lymphocytes are activated, leading to the production of short-lived plasma cells, followed by their proliferation and differentiation into memory B-lymphocytes and long-lived plasma cells for durable Ig production [39, 40]. Along these different steps, B-lymphocytes respond to diverse signals or survival/proliferation factors, including BAFF/APRIL, BCR, IL6, VEGF, EGF, and IGF-1 [39]. By blocking the specific receptor or neutralizing the ligand, the activation of signaling pathway is not delivered into the cell, leading to tumor cell growth and/or survival arrest. BAFF and APRIL belong to the TNF family that binds to the TNFR-like receptors transmembrane activator, particularly interacting with three receptors, calcium modulator and cyclophilin ligand interactor (TACI), B-cell maturation antigen (BCMA), and BAFFR for only BAFF [41, 42]. APRIL is produced by hematopoietic cells, particularly by osteoclasts [43]. The inhibition of BAFF and APRIL using a soluble receptor, TACI-Ig or atacicept (SeronoMerck Inc.), in a culture of myeloma cell lines causes rapid cell death [44] and inhibits myeloma growth in a coculture system with osteoclasts [45]. We used this drug in a Phase I study, in patients with MM and macroglobulinemia, with promising results, but this drug was mainly developed in dysimmune diseases [46–48].

Different mAbs against IL6 or soluble IL6R have been developed, particularly siltuximab, an anti-IL6 mAb, and tocilizumab, an antisoluble IL6R. Siltuximab has been recently registered for Castleman's disease in Europe and USA. Tocilizumab is registered for some dysimmune diseases refractory to anti-TNF worldwide and Castleman's diseases only in Japan [49].

IGF-1 represents the main cell communication factor produced by plasma cells and bone marrow stromal cells [50]. Inhibitors of IGF-1, including dalotuzumab and picropodophyllin, have been tested in cancers including early clinical phases of MM [51, 52]. However, as observed for IL6, the use of such specific inhibitors in very advanced diseases did not show any clinical benefit due to intraclonal heterogeneity, with the emergence of tumor cell independence from their microenvironment in addition to other growth factors [53].

## 3. Intracellular Targets

### 3.1. Cell Signaling Markers

The activation of the BCR is a major signaling pathway for B-lymphocyte function. The BCR is a multiprotein structure with a surface transmembrane Ig noncovalently associated with the Ig*α* (CD79A) and Ig*β* (CD79B) chains [54, 55]. Antigen binding to the BCR causes receptor aggregation and engagement of the signal transduction via the phosphorylation of the receptor's cytoplasmic tyrosine-based activation motifs (ITAMs) by recruited SRC-family kinases, including LYN, FYN, BLK, and LCK [54]. Then, the activation of phosphoinositide 3-kinase (PI3K3*δ*) mediates the conversion of phosphatidylinositol-4,5-bisphosphate to phosphatidylinositol-3,4,5-trisphosphate and ultimately recruits BTK [56]. BTK phosphorylation targets phospholipase C*γ*2 (PLC*γ*2), with activation of NF*κ*B and mitogen-activated protein kinase pathways. Antigen-independent signaling has been involved in B-CM which results in constitutive or aberrant BCR signaling, making BTK a major target for such diseases [57]. Ibrutinib (PCI-32765, Imbruvica, J&J Inc.) has been developed in B-CLL and B-cell lymphoma and is now approved for MCL and B-CLL. In a Phase II clinical study, a dramatic response rate was observed in both diseases, particularly in MCL with refractory disease (objective response rate (ORR) 68% including 21% CR) with a median PFS of 13.9 months [55]. Ibrutinib inhibits the adhesion mediated by chemokine and integrin to their microenvironment. This biological effect is associated with lymphocytosis and nodal shrinkage. This lymphocytosis decreased generally at the end of cycle 2. Tolerability was acceptable and adverse events included diarrhea (50%), fatigue (44%), nausea (38%), cough (31%), and myalgia (25%). As ibrutinib is metabolized by cytochrome P450 enzyme 3A (CYP3A), coadministration with CYP3A inhibitors or inducers can interfere with other drugs and may be responsible for some toxicity. Ibrutinib was associated with chemotherapy, bendamustine, or the CHOP-R regimen that associates cyclophosphamide, doxorubicin, vincristine, prednisone, and anti-CD20 mAbs, particularly in B-cell lymphoma [55]. Currently, ibrutinib is used in naive patients, especially for B-CLL and small lymphocytic lymphoma. It showed a high response rate of 71% including 13% CR, with estimated PFS and OS at 2 years of 96.3% and 96.6%, respectively, at the daily dose of 420 mg [58]. In MM, the overexpression of BTK varied, being more present in MM than in monoclonal gammopathy of undetermined significance (MGUS), with also some interindividual variability of the expression level. Despite this variability, ibrutinib was associated with a high response rate in patients with refractory MM [59].

Ibrutinib has promising activity in other B-CM, including atypical B-cell lymphoproliferative disorders (personal data) and dysimmune diseases. The active dose could be correlated with the expression level of the targeted molecule, and its measurement could be a guide to optimize the clinical efficacy. In addition, since this drug is also active in patients with poor prognostic factors such as p53 mutation or other acquired genetic modifications, there is a need to define new markers of interest and new therapeutic combinations including immune therapy to prolong the therapeutic response. Knowing the mechanisms of resistance, the effect on the normal B-cell compartment and other immune cells, the status of the immune response and following the residual disease may contribute to addressing the question of the optimal treatment duration, to avoid the mistakes made with IMiDs in MM [60]. Some resistance mechanisms have been studied, including NF*κ*B pathway and KRAS mutations. The effects of ibrutinib on normal immune cells begin to be studied, including IL2-inducible kinase that promotes a T helper 1 response, a depletion of the B-cell memory and long-lived plasma cell compartment, thus reducing a recall response or a new antigen-dependent response.

Several other BTK inhibitors are in clinical development, including ONO-4059 (Gilead Inc., USA) and AVL-292 (Celgene Inc., USA) which are reversible inhibitors of BTK. In addition, there are multikinase inhibitors, such as LFM-A13, which inhibits BTK and polo-like kinase (PLK), fostamatinib, which inhibits the *δ* isoform of PI3K and Syk [61], and dasatinib, initially developed as an inhibitor of tyrosine kinase for CML patients, which is also a BTK inhibitor.

The PI3 kinase (PIK)/AKT/mTOR pathway is an important signalling pathway for cellular functions, particularly growth and metabolism control. Different classes and isoforms of PI3Ks exist that are associated with large possibilities of inhibition leading to a great number of molecules inhibiting this pathway. IPI-145 inhibits PI3K *δ* and *γ*, and it was developed in hematological malignancies. BAY 80-6946 predominantly inhibits PI2K*α*, *δ* isoforms, as well as INK1117, a PI3K*α* inhibitor, and more than 30 other compounds. Among them, idelalisib (GS-1101, Zydelig, Gilead Inc.), a specific inhibitor of class I isoform p110*δ* was approved on July 23, 2014, in USA for the treatment of FL and B-CLL and B-cell small lymphocytic lymphoma [62]. This molecule is also active in other B-CM.

The combination of these kinases inhibitors with mAbs requires the evaluation of the impact of such molecules on effector cells, particularly NK lymphocytes. Ibrutinib did not inhibit complement activation or complement-mediated lysis. In contrast, ibrutinib and idelalisib strongly inhibited cell-mediated mechanisms induced by anti-CD20 mAbs, particularly the activation of NK lymphocytes [63]. In addition, idelalisib reduces T-regulator lymphocytes (T-regs) and could have a positive impact on tumor cells [64].

### 3.2. Cell Cycle, Proteasome, and Apoptosis Machinery

In cancer, proliferation depends on different proteins involved in cell-cycle regulation, particularly alterations of the cyclin-dependent kinase (CDK) CDK4/6-INK4-Rb-E2F cascade [65]. Resistance to chemotherapy is mediated by dysregulation of the cell-cycle machinery [66]. Overexpression of cyclins (e.g., cyclins D1 and E1), amplification of CDKs (e.g., CDK4/6), inactivation of critical CDK by CDK inhibitors (I) (e.g., p16^Ink4a^, p15^Ink4b^, p21^waf1^, and p27^kip1^), loss of retinoblastoma (Rb) expression, and loss of binding of CDKIs to CDKs (e.g., INK4 binding to CDKs) occur frequently in human malignancies [65]. Defects of apoptotic pathways are often observed in hematologic malignancies, involving the global repression of transcription by drugs that inhibit CDK7/9 [67]. Transcriptional CDKIs downregulate a great number of short-lived antiapoptotic proteins. This includes the antiapoptotic proteins myeloid cell leukemia-1 (Mcl-1) particularly critical in hematologic malignancies, the B-cell lymphoma extra long (Bcl-xL) and the XIAP (X linked IAP), D-cyclins, c-myc, Mdm-2 (leading to p53 stabilization), p21waf1, proteins whose transcription is mediated by nuclear factor-kappa B (NF-*κ*B), and hypoxia-induced VEGF [68].

Molecules that interfere with CDKs have been developed, either targeting a broad spectrum of CDKs or a specific type of CDK or targeting CDKs as well as additional kinases such as VEGFR or platelet-derived growth factor-R (PDGFR). More than 10 molecules have gone through clinical trials, including multi-CDK inhibitors such as flavopiridol (Sanofi-Aventis Inc.), a semisynthetic flavonoid, known as a pan-CDK inhibitor, developed in a large panel of hematological malignancies, SNS-032 (BMS-387032, Sunesis, BMS Inc.) developed in B-CLL, MM, and NHL, dinaciclib (SCH 727965, Merck Inc.) and PD0332991 (Pfizer Inc.) developed in various hematological malignancies, and R-roscovitine (seliciclib, CYC202, Cyclacel Inc.) [69]. The combinations of such inhibitors with cytotoxic agents but also with novel and targeted agents, including histone deacetylase inhibitors and PKC activators, NF*κ*B pathway modulators, and probably BTK and PI3K inhibitors, are programmed for clinical trials.

The ubiquitin proteasome pathway plays a critical role in regulating many processes in the cell, which are important for tumor cell growth and survival. Bortezomib was the first clinical success in some cancers and has prompted the development of the second generation of proteasome inhibitors. The ubiquitin proteasome system represents the major pathway for intracellular protein degradation, with a complex mechanism involving at least six components: ubiquitin (Ub), the Ub-activating (E1), a group of Ub-conjugating enzymes (E2), a group of Ub ligases (E3), the proteasome, and the deubiquitinases, a process that is highly controlled in normal cells, but frequently dysregulated in cancers [70].

Chemotherapy designed cytotoxic drugs which are active through impairing mitosis or fast-dividing cells by various mechanisms including damaging DNA and inhibition of the cellular machinery involved in cell division. The number of dividing cells is estimated by the mitotic index, the presence of Ki-67 positive cells on tumor samples, or the percentage of cell cycling in S phase. Such analysis may guide the prescription of cytotoxic drugs, particularly for cancers with variable percentage of cycling cells like in MM with high proliferative index superior to 4% of cells in S phase [71]. The inhibition of NF*κ*B activity modified the degradation of cell cycle-related molecules and perturbed proapoptotic and antiapoptotic protein balance, endoplasmic reticulum stress and inhibited angiogenesis and DNA repair, all the mechanisms that contribute to apoptosis of tumor cells. NF*κ*B that is constitutively active in a large proportion of cancers and is bound to its inhibitor I*κ*B within the cytoplasm, and inhibition of proteasome activity prevents degradation of I*κ*B and subsequent activation and translocation of NF*κ*B to the nucleus. Proteasome inhibitors may induce cell cycle arrest by interfering with the degradation of cell cycle regulators including cyclins. There are several inhibitors of proteasome that are used in clinic for hematological malignancies, particularly for MM and MCL, and used in combination with different other drugs such as IMiDs, other cytotoxic molecules, and dexamethasone. Major proteasome inhibitors include bortezomib, carfilzomib, but also NPI-0052, a *β*-lactone derived from the marine bacterium* Salinispora tropica*, MLN9708, CEP-18770, ONX0912, or inhibitor of the immunoproteasome such as ISPI-101 or PR-924 [70].

Apoptosis is a common process of cell death for all multicellular eukaryotic organisms leading to the eradication of damaged or infected cells. Apoptosis is initiated by two signaling pathways, the intrinsic or mitochondrial pathway and the extrinsic or death receptor pathway, that is, Fas/CD95 that binds to specific cell surface receptors. The intrinsic pathway, with members of BCL2 family, is more commonly perturbed in lymphoid malignancies, including mutation of the tumor suppressor gene TP53, which normally acts to activate certain BH3-only proteins, and the overexpression of BCL2 [72].

Obatoclax (GX15-070) is a pan-BCL2 family inhibitor, binding to BCL2, BCLx_L_, BCLw, and MCL1. Therapeutic response with obatoclax in clinical trials has been reported to be low and its development has been halted [68]. The natural product gossypol and its synthetic derivative AT-101 bind to BCL2, BCLx_L_, and MCL1 with clinical activity only when combined to rituximab for FL [73]. Antiapoptotic BCL2 proteins antagonize death signaling by heterodimer formation through binding at the BH3 domain of the protein. New molecules, BH3 mimetics were designed to functionally antagonize BCL2 protein family [74]. ABT-737 and its orally available analogue ABT-263 (navitoclax) bind and inhibit BCL2, BCLx_L_, and BCLw with high affinity, and it is developed in clinical phases, as well as ABT-199 which may be considered as the most active drug in the BCL2 family inhibitors. ABT-199 has shown high response rate (87%) in relapsed/refractory B-CLL, including bulky disease, fludarabine-refractory disease, and del17p patients [75], as well as for FL, Waldenström's disease, and MCL [73]. ABT-199 induces apoptosis within 8 hours and the most significant dose-limiting toxicity is tumor lysis syndrome. In addition, ABT-199 may be combined to chemotherapy, demethylating agents, histone deacetylase inhibitors, and novel targeted drugs such as ibrutinib and idelalisib [76].

### 3.3. Metabolic Process

In the early twentieth century, Warburg first discovered that cancer cells preferentially consume glucose and metabolize it to lactate in the presence of oxygen, named aerobic glycolysis [77]. Accumulated evidence was made to support that this metabolic way was predominant for hematological malignancies in leukemias and T-cell lymphoma, with both inducers of Warburg effect, PKM2, and HIF-1*α*, reported to be involved in AML and connected to epigenetic control of gene expression [78, 79]. This metabolic process facilitates cancer progression by resisting induction of apoptosis and promoting tumor metastasis or independence of the cancer cell microenvironment. Hypoxia is a major factor that contributes to the Warburg effect, for rapid energetic production for the cancer cell, a process favored by changes within the microenvironment. Blocking glycolysis causes a rapid dephosphorylation of BAD protein at Ser112, leading to BAX localization to mitochondria and important cell death, also observed in multidrug resistant cells [80]. The uptake of fluorodeoxyglucose positron emission tomography in cancers demonstrates the key role of glucose in the proliferation of cancer cells [81]. The generic drug dichloroacetate is a small orally available molecule known to block the pyruvate dehydrogenase kinase. It has thus been proposed in various cancers including rare patients with hematological malignancies and its use was associated with some success [82]. Through the reduction of SIRT1, the inhibition of LDH-A provides a way of altering p53 acetylation status and the downstream induction of p53 target genes selectively in cancer cells [83]. Other target is represented by peroxisome proliferator-activated receptor (PPAR), a group of nuclear receptor proteins that function as transcription factors regulating gene expression. PPAR-*α* is particularly implicated in lipid and lipoprotein metabolism and inflammation. Fenofibrate, a PPAR-*α* agonist, has been shown to induce apoptosis on certain cancer cells via activation of NF-*κ*B pathway [84]. Inhibitors for PPAR-*γ* may enhance the activity of radiation therapy in cancer [85].

There are several compounds that modulate glycolytic metabolism. This includes 2-deoxyglucose that inhibits phosphorylation of glucose hexokinase (HK), lonidamine, that inhibits glycolysis and mitochondrial respiration, HK, 3-bromopyruvate that inhibits HK and acts as an alkylating agent, imatinib that inhibits bcr-abl tyrosine kinase but also decreases HK and G6PD, and oxythiamine that inhibits pyruvate dehydrogenase [86, 87]. Specific LDH inhibitors have been developed, including AT-101, FX-11, galloflavin, N-hydroxyindole-based molecules [88], or new molecules in development by different companies. Such new molecules represent a new potent way to modulate or prevent chemoresistance. In addition, they may have some impact on immune cells [89].

## 4. Targeting Microenvironment

### 4.1. Immune Therapy

The tumoral microenvironment, and particularly immune cells, is involved in the tumor cell control or expansion. Since many years, it has been recognized that T-infiltrating lymphocytes (TIL), a mixture of different cells (Treg, T helper, T cytotoxic cells, etc.) when expanded* ex vivo*, may support some clinical efficacy [90, 91]. Nowadays, the different cell subpopulations associated with a particular function (i.e., facilitating or repressing tumor cells) may orientate the clinical prognosis and the response to therapeutic agents [92–98]. Targeting cancer cells via the immune system depends on the presence of effector cells that recognize and kill cancer cells. Recognition may be specific for adaptative response, that is, cytotoxic T-cells via antigen presentation. In the context of innate response, there are other mechanisms to recognize stress cells or non-self-cells, including activating and inhibiting molecules shared by NK, NKT, and T*γδ* lymphocytes [99]. Such cancer cell recognition may be forced by using chimeric antigen cells (CAR-T cells, CAR-NK cells) [20] or bispecific mAbs. Beyond recognition, target accessibility and tumor infiltration, mechanisms, and efficacy of killing are other criteria of efficacy to be considered. Effector cells could be directly used as cell-drugs or immune modulators that activate such specific activity, including Toll-receptor agonists [100], enhancers of ADCC and antigen presentation via dendritic cells [101], and stimulator of T*γδ*, particularly *γ*9*δ*2 T-cell, that may be purified for cellular therapy programs and activated by IL2 and bisphosphonates or IPH101 ([102–104] and personal data) combined with anti-CD20 mAbs [93]. It is surprising that using GM-CSF in addition to rituximab or IPH101 plus IL2 and rituximab, in relapsed or refractory FL, we observed similar results with 45–50% of ORR ([105] and personal data), meaning that optimal strategy is probably the direct administration of these effector cells. Development of NK cells is now one major way for immune therapy probably by using banked, activated, and amplified NK cells from cord blood samples ([106, 107] and personal data). In that way, it is important to know the efficacy of killing. For NK cells,* in vitro* data showed that one NK cell may kill 8–10 tumoral cells. Conversely, 10 cytotoxic T-cells are needed to kill one tumoral cell. This shows that NK-cell drugs are more efficient for killing, with a clinical efficacy ranging between 10^7^ and 10^9^ tumoral cells. But cytotoxic T-cells may retain a certain memory of killing and prolong the effect. This means that clinical use of such cell-drugs has different clinical targets and could be associated for a better clinical benefit. We need to simplify the therapeutic strategies and think about best combinations of drugs, cell-drugs, modifiers, and new targeted therapies.

### 4.2. Niche Disruption

Lymph node microenvironment includes different types of lymphocytes and stromal cells necessary to the antigen presentation and the education of B-cells to secrete specific antibodies. Plasmablasts generated in germinal centers exit the lymphoid organs into the lymph and then the blood, before migrating to the bone marrow or mucosa-associated lymphoid tissues where they represent a long-lived population of plasma cells in a favorable microenvironment, named plasma cell niche. Different cells constitute this niche, particularly mesenchymal cells that produce chemokines, particularly CXCL12, and bring together other niche cells (megacaryocytes, platelets, and eosinophils) and plasma cells, which all express the CXCL12 receptor, CXCR4 [108]. Within the niche, plasma cells are activated by adhesion molecules and stimulated by several survival/growth factors [109]. The hypoxic microenvironment plays a central role in controlling stem cell phenotype and dissemination, through different factors particularly the hypoxia-inducible factor-1*α* (HIF-1*α*), a key transcriptional factor that responds to hypoxic stimuli [110]. HIF-1*α* is constitutively expressed in some B-cell malignancies and is regulated by the PI3K/AKT pathway [111].

Anti-CXCR4 or CXCL12R (plerixafor and others), anti-CCR5 or CCL5R (maraviroc), inhibitors of survival/proliferation factors, that is, IL6, BAFF/APRIL, and others, but also inhibitors of osteoprotegerin, and a receptor for both RANKL and TNF-related apoptosis-inducing ligand/Apo2 (TRAIL) may represent new targets for cancer therapy [49, 112, 113]. The complex CXCL12/CXCR4 is implicated in biological mechanisms of several B-cell malignancies, particularly for CLL, MM, and lymphoma [112]. Plerixafor/AMD3100 disrupts the B-CLL microenvironment interactions, representing additional treatment, possibly with novel targeted drugs [114].

Syndecan-1 is a member of the heparan sulfate (HS) proteoglycans that are present on the cell surface or as soluble molecules shed from the cell surface. Syndecan-1 accumulates survival factors within the microenvironment, representing a sort of sponge for these factors around the tumor cells. Syndecan-1 is cleaved by heparanase, an endo-*β*-o-glucuronidase, secreted by osteoclasts [36]. As heparin and low molecular weight heparin have been known since a long time to exhibit potent antiheparanase activity, one can explain that such molecules may have a clinical impact on the cancer [115].

A new therapeutic era is born for new reflection, new methodologies and, nowadays, nearly all therapies are targeted as long as we understand biological processes for a better use of old and new drugs to support personalized medicine.

## Figures and Tables

**Table 1:** Clinical trials for B-cell chronic lymphocytic leukemia (B-CLL), lymphoma (NHL = non-Hodgkin's lymphoma), and multiple myeloma, based on research made by using key words for the different diseases, through https://clinicaltrials.gov/, as of March 13, 2015. VEGF: vascular endothelial growth factor; EGFR: epithelial growth factor; IGF-1 R: insulin growth factor receptor type 1; BTK: Bruton's tyrosine kinase; PI3k: phosphoinositide 3-kinase; HDAC: histone deacetylase; CAR-T: chimeric antigen receptor-T lymphocytes; Cdk: cyclin-dependent kinase; DKK: Dickkopf-related protein; ADCC = antibody-dependent cell cytotoxicity; CDC = complement-dependent cytotoxicity; A = direct apoptosis; M = mouse; H = humanized; Ch = chimeric; C = cytotoxicity; Phag. = phagocytosis; Doxo. = doxorubicin; Cytotox. = cytotoxicity.

		Type of mAb biological activity	Number of studies		
			Lymphoma 2246 studies	B-CLL 1965 studies	Multiple myeloma 1908 studies

Monoclonal antibodies	Anti-CD19		65	34	3
	Blinatumomab CD19/CD3				
	Anti-CD20		1017	329	20
	*Rituximab (CLL, NHL) *	Ch IgG1 ADCC, CDC, A			
	*Ofatumumab (CLL) *	HIgG1 CDC+	58	57	—
	*Obinutuzumab *	ADCC+	19	26	—
	*Ocrelizumab *	ADCC+	3	1	—
	*Veltuzumab *	ADCC, CDC	7	1	—
	*Ibritumomab tiuxetan (NHL) *	MIgG1			
	*Tositumomab (naked or * ^131^ *I) *	HIgG1	40	5	
	*NHL *				
	Anti-CD22		16	21	—
	*Epratuzumab (naked or * ^90^ *Y) *	HIgG1 trogocytosis			
	*Epratuzumab immunotoxin *				
	Anti-CD25, *LMB-2 *	Anti-TAC(Fv)PE38, C	2	—	—
	Anti-CD38 daratumumab	HIgG1, ADCC, CDC, A	—	—	6
	Anti-CD40		11	19	20
	*Dacetuzumab *	HIgG1, ADCC, phag.			
	Anti-TRAIL 2*conatumumab *	Agonist HIgG1, A			
	Anti-CD45		7	9	5
	BC8 ^131^I/BC8 ^90^Y	MIgG1			
	Anti-CD74 hLL1* milatuzumab *		1	4	2
	* (+doxo) *	HigG1, A/Cytotox.			
	Anti-CD80 galiximab	HIgG1, ADCC	4	1	—
	Anti-CTLA4 ipilimumab	HIgG1, ADCC	7	14	3
	Anti-PD-1 nivolumab	HIgG4	2	9	1
	Pidilizumab	HIgG1	—	1	1
	Anti-VEGF (sorafenib,		21	6	13
	bevacizumab)	CIgG1			
	Anti-IGF-1R	HIgG1	1	1	3
	Anti-IL6, *siltuximab, *and	CIgG1			7
	*tocilizumab *	HIgG1	—	—	2

BTK inhibitor	*Ibrutinib *		9	47	3
	*spebrutinib, *and*ONO-4059 *				

PI3 kinase/Akt/mTOR/PIM/MEK inhibitor	Idelalisib, duvelisib, and TGR-1202		23	14	8
	RP6530 (dual PI3K*δγ*)				
	MK2206, AMG 319				
	LGH447, BYL719				
	Pictilisib (GDC-0941)				
	GSK1120212, GSK110183				
	Nelfinavir, CUCD-907				

Proteasome inhibitor	Ixazomib, salinosporamide,		20	6	77
	Filanesib, oprozomib, and lapatinib				

HDAC	Vorinostat, ricolinostat,		135	26	56
	panobinostat, givinostat,				
	4SC202, entinostat,				
	quisinostat, rocilinostat,				
	tacedinaline, abexinostat, and				
	CDX101				

CAR-T	CD19, CD30, CD20, CD22, and CD138		13	24	3

Other drugs	Somatostatin analog				1
	(pasireotide)				1
	Anti-DKK1		13	6	5
	CDK inhibitors				
	Anti-EGFR (erlotinib,		2	—	—
	crizotinib)				

**(a):** 

Follicular lymphoma 1031 studies	Monoclonal antibodies 232 studies	Anti-PD-1	2 studies	Combination with rituximab
		CD20 radio	34 studies	Combination
		immunotherapy	including	+ ASCT
			Phase 3	
		CD45 ^131^I	1 study	+ ASCT
			Phase 2	
		Anti-CTLA-4	1 study	Combination with SD-
				101 (TLR9 agonist)
		Anti-CD20	10 studies	+ lenalidomide
			Phase 1	maintenance
			Phase 2	
		Bevacizumab	Phase 1	Combination
			Phase 1	+ vandetanib
		Apolizumab	Phase 1	
		(anti-DR)	Phase 2	
		Galiximab (anti-CD80)	Phase 2 1 study	+ rituximab
		Anti-CD19	Phase 1	
			2 studies	
		Anti-CD22	Phase 1/2	+ ASCT
		Radioimmunotherapy	Phase 2/3	
		Cold	Phase 1	
		Anti-CD74	Phase 1/2	Combination with rituximab
		Anti-CD20+IL12		
		Anti-*α*v*β*3 integrin	Phase 1	
		Anti-CD80	Phase 1/2	
		BMK120	Phase 1	Rituximab
		Buparlisib		
	PI3K inhibitor	BAY80-6946	Phase 2	
		Idelalisib	Phase 3	Combination
		Entospletinib		Phase 1
	BTK inhibitor	Ibrutinib/ONO 4059 Spebrutinib	Phase 2	10 studies
	Anti-CDK	Flavopiridol	Phase 1	Combination
		Antisense	Phase 2	Combination with rituximab
	Anti-Bcl-2		2 studies	
		Obatoclax	Phase 1/2	
	Anti-PARP	Alisertib Veliparib	Phase 2 Phase 1/2	Combination
	HDAC	Vorinostat	Phase 2	+ rituximab
	Anti-kinase	Vandetanib	Phase 1	

**(b):** 

		Anti-CD20		
		Rituximab	Phase 2	Chemo, vorinostat,
			Phase 3	bortezomib
		Ofatumumab	Phase 1	
		Ublituximab ^90^Y/^131^I		+ lenalidomide
				maintenance
				+ ASCT
		51 studies		
		Anti-CD56 ^131^I		
		3 studies	Phase 1	+ ASCT
				
		Anti-VEGF		
	Monoclonal antibodies 158 studies	bevacizumab	Phase 2	3 studies
		Anti-VEGF kinase		
		(cediranib)	Phase 1	+ bevacizumab
		Anti-transferrin R	Phase 1	
		Anti-CTLA4	Phase 1/2	4 studies
		Anti-HLA DR	2 studies	Phase 1
		Anti-CD22	Phase 1	1 study
		Anti-CD22 ^90^Y	Phase 1/2	+ anti-CEA In111 1 study
Mantle cell lymphoma 860 studies		Anti-CD22 In111	Phase 1/2	
		Anti-*α*-v *β*3 integrin	Phase 1	
		Anti-CD19	Phase 1/2	
		Anti-CD74	Phase 1/2	+ veltuzumab (humanized MoAb)
		Anti-IGF-1R	Phase 1/2	
		ganitumab		
		Anti-TRAIL R2	Phase 1	+ bortezomib/vorinostat
		conatumumab		
	Anti-PI3K	Idelalisib	Phase 1	Chemo/rituximab
		BKM120	Phase 1	+ rituximab
	Anti-BTK	Ibrutinib	Phase 1	Chemo/rituximab
	Anti-cdk	Flavopiridol	Phase 1	+ chemo/rituximab
	mTOR inhibitor	Temsirolimus	Phase 2	+ rituximab
			Phase 1/2	+ cladribine/rituximab
	Anti-endosialin/TEM1		Phase 1	
	HDAC	Romidepsin	Phase 1/2	+ rituximab/lenalidomide
	Anti-bcl2	Oblimersen	Phase 2	+ rituximab
		Obatoclax		+ chemo/rituximab
	Aurora-kinase inhibitor	Alisertib	Phase 2	+/− rituximab
	Dehydrogenase inhibitor	CPI-613	Phase 1	+ bendamustine/rituximab
	HDAC	Vorinostat	Phase 1/2	+ chemo
	Toll-R agonists	CPG 7909	Phase 2	+ chemo

**Table 3:** Clinical trials for multiple myeloma, based on https://clinicaltrials.gov/, as of March 13, 2015. ASCT: autologous stem cell transplantation; PD-L-1: programmed death-1 ligand 1; CTLA-4: cytotoxic T-lymphocyte-associated protein 4; IGF-1R: insulin growth factor-1 receptor; KIR: killer cell Ig-like receptor; DKK: Dickkopf-related protein; BTK: Bruton's tyrosine kinase; PI3k: phosphoinositide 3-kinase; PARP: poly(ADP-ribose) polymerase.

Multiple myeloma 1908 studies	Monoclonal antibodies 82 studies	Anti-CD38	4 studies	Phase 2
		Anti-IL6 siltuximab	5 studies	Combination
		Anti-CD40	4 studies	Phase 1/2
		Anti-transferrin R	1 study	Phase 1/2
		Anti-GM2	1 study	Phase 1/2
		Anti-CD66 ^90^Y	1 study	+ ASCT
		Anti-CD45 ^90^Y	1 study	+ allogeneic transplantation
		Anti-Adhesion Mol1	1 study	Phase 1
		Anti-CD38	1 study	Combination
		Anti-PDL1	3 studies	Phase 2 with vaccination
				Combination with lenalidomide
		Anti-IGF1R	1 study	Phase 1
		Bevacizumab	3 studies	Phase 2 combination
		Anti-KIR	4 studies	Phase 1 and Phase 2
		Anti-CTLA-4	2 studies	+ allogeneic T.
		Anti-CD52	3 studies	+ allogeneic T.
		Anti-DKK1	1 study	Randomized Phase 2
		Anti-CD20	^90^Y/^131^I 2 studies	+ ASCT
			Cold: 3 studies	Combination
		Anti-CD56	1 study	Phase I
		Elotuzumab	5 studies	Randomized Phase 1/2
		Anti-GRP78 (PAT-SM6)	1 study	Phase 1
		Anti-CXCR4	1 study	Phase 1b
	BTK inhibitors	Ibrutinib ACP-196/ACP-319	2 studies (+ carfilzomib) 3 studies	Phase 2 Phase 1b
	PI3 kinase inhibitors	Idelalisib, BYL719, CUDC-907, nelfinavir, SOM230 LAR, and sorafenib		Phase 1/2
	Anti-CDK	Dinaciclib	Combination	Phase 1/2
	Antiproteasome	Carfilzomib Ixazomib Oprozomib Marizomib	10 studies	Phase 3 Phase 1
	HDAC	Ricolinostat, vorinostat	Combination	Phase 1b
	PARP inhibitor	ABT-888	Combination	Phase 1
